# Stable hemoglobin in hemodialysis patients: forest for the trees – a 12-week pilot observational study

**DOI:** 10.1186/1471-2369-14-243

**Published:** 2013-11-04

**Authors:** Jacques B Rottembourg, Floride Kpade, Fadia Tebibel, Aurélie Dansaert, Gaelle Chenuc

**Affiliations:** 1 Dialysis Unit, Diaverum Group, Clinique Mont Louis, 11, passage Courtois, 75011, Paris, France; 2 Cegedim Strategic Data, Boulogne Billancourt, France

**Keywords:** Darbepoetin alfa, Hemodialysis, Hemoglobin cycling, Inter-dialysis complications, Per-dialysis events

## Abstract

**Background:**

Hemoglobin (Hb) variability is a common occurrence in hemodialysis patients treated with erythropoiesis-stimulating agents. High amplitude fluctuations have been associated with greater risk of morbidity and mortality.

**Methods:**

This prospective, single centre pilot observational study was conducted over a 3-month period in daily practice patterns, to assess per-dialysis events and inter-dialysis complications that could interfere with erythropoiesis in patients undergoing hemodialysis.

**Results:**

Mean Hb levels remained stable in the 78 evaluable patients, as did darbepoetin alfa (DA) doses, including in patients suffering from diabetes or cardiac affections. In total, an average of 7.7 events / patient / month occurred, but no significant relationship with Hb excursions was shown.

**Conclusion:**

The observation of 7.7 events per patient per month suggests a careful monitoring of Hb and DA dosing every other week, in order to maintain Hb level within the target.

## Background

Anemia is an important and common complication in patients with end-stage renal disease treated with chronic maintenance hemodialysis (HD). Its impact on cardiovascular outcomes, quality of life and even survival has been widely assessed. As a result, throughout the years, several guidelines have defined the targets in hemoglobin (Hb) levels that need to be achieved in HD patients [1-5]. These have been recently modified by the KDIGO clinical practice guidelines for anemia in chronic kidney disease (CKD) [6]. Treatment with erythropoietin stimulating agents (ESAs) has been a major advance in the management of this problem. There is, however, a great difference between treatment and physiological erythropoietic biology, in so far as ESAs are administered episodically, generating important changes in erythropoietin – and hence – Hb levels [7]. This phenomenon, which is commonly encountered in ESA-treated patients [8,9], is known as Hb cycling. It can be defined as cyclical increases and decreases of measured Hb levels in individual patients [7]. Its consequences are not fully known, however it may be accepted that Hb cycling can result in fluctuations of the oxygen carried to the tissues, and repeated episodes of relative ischemia in vital organs can lead to organ dysfunction or injury. Indeed, high amplitude swings have been found to be associated with greater risk of mortality and hospitalization [10].

Apart from anemia management, several factors can be accounted for causing Hb cycling: amongst others, inflammatory and infectious diseases, blood loss, hyperparathyroidism and hospitalizations [7-9]. On the other hand, little is known about dialysis-related incidents, whether they occur during or between HD sessions.

This prospective, single-center pilot observational study was conducted over a 3-month period, to assess the number and importance of per-dialysis events and inter-dialysis complications that could possibly interfere with erythropoiesis in patients undergoing hemodialysis.

## Methods

Patients were treated according to routine clinical practices in the unit. As required by law, no formal approval was required by the independent ethic committee, and patients provided written informed consent prior to study entry [11]. All patients treated in our center were included for 12 weeks in the study. They were prescribed regular HD on Integra-Hospal generators for 3.5 to 4 hours three times weekly. Vascular access was assessed by double arterio-venous fistula punctures in all patients. On start of the dialysis session, each patient received a 0.2 to 0.6 ml injection of nadroparin calcium, a low molecular weight heparin. High and medium permeability membranes were used throughout the study and were unchanged for each patient. Darbepoetin alfa (DA) injections were performed every other week (during the first dialysis session of the week) in the venous injection site before the drip chamber on the venous line (Pivipol Hospal). Any change in the dosing of DA required a follow-up of four Hb measurements, and the adaptation was made by one physician who knows the previous doses and the clinical program of the patient: if, on 4 Hb values, one was outside of the target, nothing was changed; if 2 or 3 values were outside of the target, the dosing was adapted depending the amplitude. Intravenous (IV) iron (iron sucrose [V]) was injected, if necessary, once a week during the second dialysis session at a dose ranging from 25 to 100 mg.

The follow-up conditions were those of daily practice, and the protocol ensured that no complementary visits or biological assessments would be performed outside the usual HD follow-up. Biological parameters such as Hb, calcium, phosphorus, urea, creatinine and DA dose, were assessed every other week; iron dose was recorded every week. Other parameters, and especially C-reactive protein, PTH and nutritional parameters [albumin and normalized protein catabolic rate (nPCR)] were assessed at baseline and on week 12; dialysis adequacy was assessed at the same period by Kt/V and urea reduction ratio (URR). Hb variation was defined as a ≥ 1 g/dl Hb excursion above or under baseline value and a ≥ 4 week-duration.

Upon each dialysis session, per-dialysis events, such as fistula bleeding, puncture problems, hypotension, clotting in lines or dialyzer, blood loss, and delayed coagulation, were recorded by the nurses on specific questionnaires. Physicians assessed each week inter-dialysis complications, mainly cardiovascular, digestive, infectious, hemorrhagic, and vascular access, by three means of consultation: face to face, by phone, and, when in doubt, by consulting the family.

Descriptive statistics were applied for quantitative variables, and results are reported with mean values and standard deviations; median values and ranges are also provided when appropriate, owing to dispersion of values. A uni-variate analysis was performed to assess if there is an association between events (per-dialysis and inter-dialysis) and hemoglobin excursion. The evaluable population comprised patients who attended every dialysis session during this prospective study; patients who were absent for any reason (vacation, prolonged hospitalization, graft, death) were excluded.

## Results

### Evaluable population

Out of 100 patients undergoing HD in the center, 78 attended every dialysis session for 3 months, and therefore constituted the evaluable population. 22 patients were excluded because they did not attend all the dialysis sessions: 2 patients died, 4 were grafted, 7 were hospitalized for 3 to 28 days, and 9 went in vacation. The demographics and characteristics of these 78 patients, at baseline, were shown in Table 1. More than two-thirds were males, and the mean (± SD) age was 59 (± 16.6) years. They had been undergoing dialysis for a median time of almost three years (34.5 months). Nearly all of them (94.9%) suffered from hypertension, and 47.4% from cardiac insufficiency. Medical history and current treatments at baseline are shown in Table 2.

**Table 1:** Demographics and characteristics at baseline

**Characteristics**	**Evaluable patients**
	**(n = 78)**
**Gender, n (%)**	
Male	54 (69.2)
Female	24 (30.8)
**Age (years)**	
Mean (SD)	59.0 (16.6)
**BMI (kg/m**^**2**^**)**	
Mean (SD)	22.6 (4.3)
**Primary cause of renal failure, n (%)**	
Diabetes	16 (20.5)
Hypertension	10 (12.8)
Glomerulonephritis	24 (30.8)
Other	28 (35.9)
**Dialysis vintage (months)**	
Mean (SD)	49.6 (47.1)
Median (range)	34.5 (2.0-293.0)

**Table 2:** Current history and medical treatments at baseline

**Characteristics**	**Evaluable patients (n = 78)**
**Medical History, n (%)**	
Hypertension	74 (94.9)
Cardiac insufficiency	37 (47.4)
NYHA II	6 (16.2)
NYHA III	25 (67.6)
NYHA IV	6 (16.2)
Coronary artery disease	22 (28.2)
Myocardial infarction	5 (6.4)
Stroke	7 (9.0)
Limb arteritis	27 (35.5)
Hepatitis	20 (25.6)
Cancer	10 (12.8)
**Current treatments, n (%)**	
ACE inhibitors and/or ARBs	23 (29.5)
VKAs, aspirin, platelet aggregation inh.	37 (47.4)
Dyslipidemia treatment	61 (78.2)
Hepatitis treatment	7 (9.0)
Cancer treatment	17 (21.8)

NYHA: New York Heart Association; ACE: angiotensin converting enzyme; ARBs: angiotensin receptor blockers; VKAs: vitamin K antagonists.

### Hemoglobin and DA dose, iron dose

**Figure 1:**
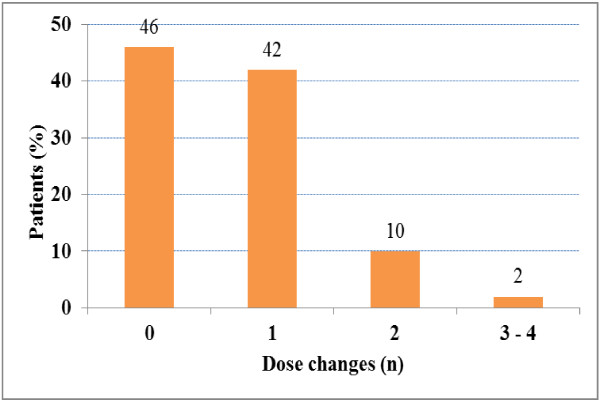
Proportions of patients requiring DA dose changes over time.

Over the study period, mean Hb levels remained stable in the evaluable population, as did DA doses (Table 3). Throughout the study, approximately 80% of patients had levels ≥ 11 g/dl. Similar findings were reported for patients suffering from cardiac affections or from diabetes (Table 4). A vast majority of patients required very few, if any, changes in DA doses during the 3-months duration of the study: 46% of patients did not require any change, whilst 42% only required one change (Figure 1). Twelve patients require more than one adaptation of DA doses during the study: these patients are the one who suffered of the most events. Seven patients did not receive any DA treatment; as a whole, their Hb levels were slightly higher than those of the patients receiving DA: median Hb was 12.0 g/dl at baseline, and remained stable over the 3-month period, even rising to a median of 13.2 g/dl in the last two weeks. Sixty eight patients received IV iron at a mean (± SD) dosage of 59 (± 35) mg per week.

**Table 3:** Hemoglobin concentrations and DA doses over time - Evaluable population (n = 78)

**Variables**	**Baseline**	**W2**	**W4**	**W6**	**W8**	**W10**	**W12**
**Hemoglobin (g/dl)**							
Mean (SD)	11.8 (1.34)	11.8 (1.25)	11.9 (1.27)	11.9 (1.43)	11.8 (1.57)	12.0 (1.35)	11.8 (1.47)
Median	11. 7	11.6	12.1	11.8	11.7	11.9	11.8
≥ 11 g/dl (%)	79.5	79.2	82.1	78.1	73.0	81.9	80.5
**Weekly DA dose (μg/week)**							
Mean (SD)	47.7 (40.2)	45.9 (40.8)	45.6 (40.8)	43.2 (39.6)	42.7 (39.3)	42.2 (39.6)	40.9 (38.5)
Median	30	30	30	30	30	30	30
**Weighted weekly DA dose**							
**(μg/week/kg)**							
Mean (SD)	0.76 (0.7)	0.75 (0.8)	0.74 (0.8)	0.70 (0.7)	0.70 (0.7)	0.70 .8)	0.67 (0.7)
Median	0.48	0.46	0.47	0.42	0.42	0.41	0.39

**Table 4:** Hemoglobin concentrations and DA doses over time – Cardiac and diabetic patients

**Variables**	**Cardiac patients (n = 46)**			**Diabetic patients (n = 21)**		
	**Baseline**	**W6**	**W12**	**Baseline**	**W6**	**W12**
**Hemoglobin (g/dl)**						
Mean (SD)	11.9 (1.5)	12.1 (1.5)	11.7 (1.7)	12.0 (0.7)	11.9 (0.8)	11.7 (1.0)
Median (range)	11.9 (6.8-15.2)	11.9 (9.3-17.4)	11.8 (6.4-17.0)	11.9 (11.0-13.2)	11.7 (10.6-14.0)	11.8 (9.5- 13.2)
**Weekly DA dose**						
**(μg/week)**						
Mean (SD)	52.9 (44.0)	44.7 (40.1)	43.3 (39.5)	38.6 (33.9)	35.3 (33.0)	29.5 (22.1)
Median (range)	40.0 (10.0-200.0)	30.0 (10.0-200.0)	30.0 (10.0-200.0)	30.0 (10.0-150.0)	25.0 (10.0-150.0)	20.0 (10.0-100.0)
**Weighted weekly DA dose**						
**(μg/week/kg)**						
Mean (SD)	0.8 (0.7)	0.7 (0.7)	0.7 (0.7)	0.5 (0.5)	0.5 (0.5)	0.4 (0.3)
Median (range)	0.53 (0.2-3.3)	0.43 (0.2-3.3)	0.45 (0.1-3.3)	0.38 (0.2-2.4)	0.38 (0.2-2.4)	0.36 (0.1-1.5)

### Biological variables

Iron status remained stable throughout the study (Table 5). Serum ferritin was ≥ 100 μg/l in almost 95% of patients, both at baseline, and at the end of the study. C-reactive protein at baseline, week 6 and week 12 was < 10 mg/l in a majority of patients (76.2%, 84.5.0% and 89.8% respectively). During the 3-month study period, nutritional variables also remained stable in the evaluable population (Table 5), as well as in cardiac and diabetic subgroups. Dialysis adequacy was achieved with a mean (SD) urea reduction ratio between 0.73(0.07) at baseline and 0.70 (0.07) at the end of the study and Kt/V at 1.49 (0.39) and 1.35 (0.34) respectively. Serum calcium, phosphorus and PTH were maintained in the current guidelines.

**Table 5:** Biological variables over time – Evaluable population (n = 78)

**Variables**	**Baseline**	**Week 12**
**Ferritin (μg/l)**		
Mean (SD)	478 (289.4)	468 (265.4)
Median	456	443
< 100 μg/l, n (%)	4 (5.1)	4 (5.2)
**Transferrin saturation (%)**		
Mean (SD)	40 (9.2)	41 (8.9)
Median	41	41
**Albumin (g/l)**		
Mean (SD)	38.9 (4.4)	40.7 (4.5)
Median	38.7	40.7
**nPCR (g/kg/day)**		
Mean (SD)	0.9 (0.2)	0.8 (0.2)
Median	0.9	0.8
**C-reactive protein (mg/l), n (%)**		
Mean (SD)	9.8 (13.5)	6.4 (10.3)
< 10 mg/l, mean(SD), (%)	4.4 (2.0), (76.4)	4.1 (1.8), (89.9)
≥ 10 mg/l, mean(SD), (%)	27.1 (19.5), (23.6)	29.8 (25.0), (10.1)

nPCR: normalized protein catabolic rate.

### Per-dialysis events and inter-dialysis complications

Intercurrent events over the study duration, i.e. 2,808 dialysis sessions, are summarized in Table 6. As expected, most per-dialysis events were related to blood circuit, and almost all patients experienced at least once clotting (97.4%) or blood loss and delayed coagulation (93.6%). Hypotensive episodes occurred at least once in 84.6% of patients. Events related to HD technique, such as puncture problems and fistula bleeding, occurred less frequently with a total of 149 and 38 respectively, and concerned one half and one third of patients respectively.

**Table 6:** Intercurrent events during the 3-month study

**Events**	**Total number of events**	**% of patients with ≥ 1 event**	**Mean number of events/patient/month**
**Peridialysis events**			
Fistula bleeding	38	33.3	0.2
Puncture problem	149	50.0	0.6
Hypotension	308	84.6	1.3
Clotting (lines, dialyzer)	596	97.4	2.6
Blood loss & delayed coagulation	425	93.6	1.8
**Interdialysis complications**			
Cardiovascular	48	34.6	0.2
Digestive	76	59.0	0.3
Infectious	83	57.7	0.4
Hemorrhagic	52	41.0	0.2
Vascular access	20	21.8	0.1

Vascular access complications were also less frequent, occurring in 21.8% of patients. The most frequent inter-dialysis complications were digestive (76 experienced by 59% of patients), and infectious (83 experienced by 57.7%). There were no relationship between hemoglobin excursions and intercurrent events (Table 7).

**Table 7:** Intercurrent events according to hemoglobin excursion

	Hemoglobin excursion*		
	**All**	**No**	**Yes**
**Events**	**(N = 78)**	**(N = 46)**	**(N = 32)**
**Cardiovascular complication**	27 (34.6%)	17 (37%)	10 (31.3%)
**Digestive tract complication**	46 (59%)	27 (58.7%)	19 (59.4%)
**Infectious complication**	45 (57.7%)	26 (56.5%)	19 (59.4%)
**Hemorrhagic complication**	32 (41.0%)	16 (34.8%)	16 (50.0%)
**Access complication**	17 (21.8%)	12 (26.1%)	5 (15.6%)

*Hemoglobin excursion: variation ≥1 g/dl in up or down and duration ≥4 weeks.

In total, an average of 7.7 events / patient / month occurred, that might interfere with erythropoiesis. There is no correlation between per-dialysis events and hemoglobin excursions (r = 0.18, p < 0.52) and inter-dialysis events and hemoglobin excursions (r = 0.21, p < 0.51).

## Discussion and conclusions

Anemia, a common complication in patients with end-stage renal disease on dialysis has been shown to increase the risk of morbidity and increased mortality, and alters quality of life. Ebben and colleagues studied 152,846 Medicare hemodialysis patients and defined six patients groups on the basis of hemoglobin level fluctuation [10]. The consistently low group (Hb < 11 g/dl) had the highest percentage of hospitalizations and the highest number of co-morbid conditions. On the other hand, the CHOIR trial, in patients not on dialysis, showed that targeting a hemoglobin level of 13.5 g/dl was associated with a significantly increased risk of a composite endpoint of death, myocardial infarction, hospitalization for congestive heart failure, and stroke, without any significant improvement in quality of life [12]. A same figure was observed on hemodialysis in the Normal Hematocrit Study [13]. It appears that both low and high Hb levels are associated with increased mortality risk, in a U-shaped or inverse J-curve relationship [14]. Somewhat conflicting results have been published, however: in a 2-year duration study of 5,037 European HD patients, Eckard and colleagues determined Hb variability, and concluded that, although it is frequently seen in these patients, it does not independently predict mortality [15].

The findings of our study support the hypothesis that patient-related factors such as per-dialysis events, inter-dialysis events and co-morbid conditions could influence the degree of Hb variability in patients undergoing HD, if the monitoring of Hb and the dosing of DA are done with an interval exceeding two weeks. A total of 1516 per-dialysis events such as fistula bleeding, puncture problems, hypotension, clotting in the lines or dialyzer or delayed coagulation resulting in blood loss were observed along with 279 inter-dialysis events such as cardiovascular, infectious hemorrhagic and vascular access complications that did not lead to hospitalizations, or for no more than two days. This resulted in a mean 7.7 events per month per patient potentially interfering with erythropoiesis.

Defining an optimal level for Hb patients with CKD is therefore difficult. This may explain why guidelines have evolved during the past decade, recommending a target of 10 to12 g/dl for the latest KDOQI guidelines [16,17], which is narrower for ERBP (11–12 g/dl) [18], and much higher than the new KDIGO (9.5-11.5 g/dl) [6].

A small number of HD patients, however, have normal Hb levels and have no need for ESAs. A population of 45 non anemic HD patients was compared to a control group of 205 HD patients on ESA therapy [19]. Absence of anemia was more frequent in men and younger patients with long-term renal replacement therapy, in patients with HCV + liver disease and adult polycystic kidney disease, associated with increased endogenous erythropoietin production and renal and hepatic cysts. In our study, 7 of the 78 evaluable patients did not receive any ESA therapy, 3 of them had a polycystic kidney disease, 3 were diabetics and one had a nephrosclerosis.

More than a set target level, however, fluctuations in Hb levels have been shown to be associated with co-morbid situations. This frequent phenomenon, known as hemoglobin cycling, can be defined as fluctuations over time in measured Hb levels, increasing above or decreasing below the target and reversing direction [7]. Apparently stable mean Hb levels in an overall study population can hide the occurrence of intra-individual variability in many patients. In a retrospective data analysis of 281 HD patients receiving ESAs, an occurrence of Hb cycling in 90% of patients has been reported [9]. Lacson and colleagues reported important fluctuations over time, with only 5% of ESRD patients in the target range during a 6-month period observation [20]. In another retrospective study, undertaken in the Netherlands, none of the 97 ESA-treated patients had Hb levels stable within the target range over a one-year period [21]. In our present study, we report stable levels of hemoglobin as a whole in the evaluable population, as well as in the cardiac and diabetic subgroups, during the whole study period. However 54% of the patients needed at least one change in darbepoetin alfa dose, to compensate Hb fluctuations outside the target level. In our unit, over the last few years, the median number of darbepoetin alfa dose changes per year is about three [22].

Numerous causes have been identified that can result in Hb cycling. Some are patient-related, such as age (older patients show smaller variability) [23], co-morbid conditions, e.g. inflammatory states or infections [10], levels of parathyroid hormone, and concurrent hematologic disorders. In HD patients, red blood cells have a short circulating half-life, due to fluctuations in erythropoietin levels, and this is a component of variability in Hb levels [24].

Other causes of Hb variability are drug-related: ESAs have been a major advance in anemia management, but also contribute to Hb cycling, and compounds pharmacokinetics, dosing and frequency of administration generate substantial changes in erythropoiesis [25-27]. For Portoles, long-acting ESA, darbepoetin alfa, achieved better Hb stability than short-acting ESA [28]. Iron homeostasis also plays an important role in anemia management [29]. If the etiology of Hb variability is not easily discerned, erythropoietic hyporesponsiveness must be envisaged and evaluated, and all efforts brought to reaching stability [30,31].

Hb testing procedures, practice patterns and reimbursement policies can also contribute to Hb fluctuations. Publications have mentioned center-effects: a retrospective observational study conducted on the US Renal Data System (USRDS) in 2000 and 2001 identified a wide center variation in hematocrit, with a mean difference of 3.06 between the poorest versus the best performing units [32].

All these factors can thus contribute to intra-patient variability. Amongst them, intercurrent events, such as inter-dialysis complications and per-dialysis events have been little studied. De Francisco and colleagues carried out a post-hoc analysis of Hb concentrations on the pooled records of 5,592 HD patients included in Phase 3b trials on the efficacy and safety of darbepoetin alfa for the management of anemia [23]. The intra-patient variability was significantly greater in the presence of infection or inflammation, blood transfusion, or hospitalization including for cardiovascular causes. Little is known about the per-dialysis events which are the most frequent events observed in the HD population: compared to patients treated by peritoneal dialysis, the Hb level is less stable in patients treated by HD [33] and the requirement of ESAs doses much higher in the HD population [34,35]. Moreover inter dialysis hypotension episodes, which are one of the most frequent events, are known to disturb hemoglobin stability [36], and patients suffering of frequent intradialytic hypotensive episodes present a hemoglobin level lower than that of the stable patients [37].

It has been advised that strategies to minimize Hb cycling should focus on individualizing targets for the patients. Targets defined by the guidelines are convenient for most patients. However, authors have recommended that the therapeutic decision should center on what Hb is most appropriate at a “safe ESA dose”, even if it means a target of 12 to 13 g/dl [38].

In our experience, in order to achieve and stabilize Hb levels within a safe range, we use moderate DA doses and address the ongoing iron needs of the patients, now injected on the same Q2W basis than DA [39]. Monitoring both Hb and DA dosing every other week appears to be the most sensible way to achieve this goal. However, our study is a pilot study, and needs to be confirmed by wider multicenter trials.

## Competing interests

Jacques Rottembourg: No sponsorship or Funding Arrangements during the past five years. Honoraria and reimbursements for lectures from Amgen S.A.S, Fresenius GMH, Vifor LtD.

Floride Kpade: The author declares no competing interest.

Fadia Tebibel: The author declares no competing interest.

Aurélie Dansaert: The author declares no competing interest.

Gaëlle Chenuc : The author declares no competing interest.

## Authors’ contributions

JR has contributed to the conception, design, supervision of the study, interpreting the data and wrote the article. FK has contributed to the acquisition of the data. FT has contributed to the acquisition of the data. AD has contributed to the planning of the study in the dialysis unit. GC performed acquisition, analysis and interpretation of data. All authors read and approved the final manuscript.

## Pre-publication history

The pre-publication history for this paper can be accessed here:

http://www.biomedcentral.com/1471-2369/14/243/prepub
